# Silicon at the Soil–Plant–Microbiome Interface: Rhizospheric Reconfiguration and Crop Resilience to Environmental Stresses

**DOI:** 10.3390/plants15091320

**Published:** 2026-04-25

**Authors:** Aziz Boutafda, Said Kounbach, Ali Zourif, Rachid Benhida, Mohammed Danouche

**Affiliations:** 1 Department of Chemical and Biochemical Sciences—Green Process Engineering (CBS-GPE), College of Chemical Sciences and Engineering (CCSE), University Mohammed VI Polytechnic (UM6P), Ben Guerir 43150, Morocco; aziz.boutafda-ext@um6p.ma (A.B.); said.kounbach@um6p.ma (S.K.); ali.zourif-ext@um6p.ma (A.Z.); rachid.benhida@um6p.ma (R.B.); 2 Institute of Chemistry of Nice, UMR7272, Côte d’Azur University, French National Center for Scientific Research (CNRS), 06108 Nice, France

**Keywords:** silicon, fertilization, rhizosphere processes, silicate-solubilizing microorganisms, Lsi transporters, abiotic stress mitigation, biotic resistance

## Abstract

Silicon is increasingly applied in agriculture to improve plant productivity under both abiotic and biotic stress constraints. Nevertheless, its mechanisms of action are often studied separately at the soil, plant, or microbiome levels, limiting a comprehensive understanding of its overall impact on agroecosystem functioning. This review proposes an integrated perspective of the soil–plant–microbiome continuum, linking silicon chemistry in soil solutions with the effects of silicon amendments on soil properties and the processes of uptake, transport, and deposition in the plants. We show that silicon bioavailability depends on maintaining a pool of dissolved silicon dominated by orthosilicic acid, regulated by mineral weathering, adsorption–desorption dynamics, polymerization, pH, iron and aluminum oxides, and organic matter. In soils, silicon inputs can improve structure, modulate acidity and cation exchange balances, influence nutrient availability, and reduce the mobility of certain metals. They may also affect enzymatic activities and microbial community composition. In plants, silicon uptake and transport, mediated by specific transporters, contribute to tissue silicification, the maintenance of leaf architecture, and the regulation of water, ionic, and redox homeostasis. These processes provide a basis for enhanced tolerance to drought, salinity, and metal toxicity, as well as biotic stress caused by pathogens and pests. Finally, we discuss key limitations to the agronomic application of silicon, including the diagnosis of the silicic status of soils, the choice of source and mode of application, and the genotypic variability of acquisition, as well as the need for multi-site tests and more robust mechanistic validations. This synthesis provides a coherent mechanistic framework to better define the conditions under which silicon can serve as a reliable tool for sustainable crop management under climate change.

## 1. Introduction

Silicon (Si) is increasingly recognized as a beneficial element in agriculture, contributing to plant development, particularly under environmental or biological stress conditions [1]. Although not classified as essential for completing the plant life cycle under optimal conditions, extensive research indicates that Si significantly enhances plant performance when crops are exposed to environmental constraints [2]. This role is especially evident in high Si-accumulating species such as rice (*Oryza sativa*) [3], wheat (*Triticum aestivum*) [4], barley (*Hordeum vulgare*), corn (*Zea mays*) [5], and sugarcane (*Saccharum officinarum*) [6]. However, positive effects have also been reported in cucurbits and forage grasses [7,8], where tissue Si concentrations can reach up to 10% of dry mass [9], indicating their crucial role in plant performance, stress tolerance, and tissue stability [10,11].

The agronomic effects of Si are highly context-dependent. Their magnitude varies with soil properties, the chemical form and source of Si, the mode of application, plant genotype, and the nature and intensity of the stress considered [11,12,13]. This variability indicates that Si responses cannot be fully understood from plant physiology alone. Rather, they must be interpreted through an integrated framework linking Si dynamics in soil solution, soil physical–chemical–biological functioning, plant uptake and tissue deposition, and rhizosphere-associated microbiome processes [12].

Agronomically, this growing interest in Si is accompanied by an increasing diversification of sources, formulations, and application modes, which reinforces the need for more comparable recommendations across studies and better transferability to field conditions [12,13]. Current Si inputs include three major categories: soluble sources such as alkaline silicates [14,15], stabilized orthosilicic acid formulations [16,17], and solid sources such as calcium silicates (Ca–Si) or calcium–magnesium silicates (Ca–Mg–Si), wollastonite, and silicate slags or co-products [18,19,20,21,22]. These products can be applied through soil incorporation, at sowing, by fertigation, or as foliar sprays, depending on the crop and agronomic objective [13,16,23]. The reported rates also vary widely, from soil applications in rice to foliar sprays in horticultural crops [23,24]. This diversification is further reflected in the emergence of a structured commercial offer, including products such as AgSil^®^ 16H, YaraAmplix™/ACTISIL™, and AgroSilício, and in the expansion of the global Si input market, estimated at approximately 450 thousand tons and projected to reach 580 thousand tons by 2035 (CAGR 3.07%) [25]. However, this growing commercial and agronomic momentum contrasts with the still limited mechanistic integration needed to explain when, where, and why Si produces reliable benefits [13]. Despite the abundance of published work, the literature remains largely fragmented across three dimensions that only partially overlap: the soil chemistry of Si, plant uptake and deposition processes, and interactions with rhizosphere microbial communities [26,27]. This fragmentation limits mechanistic understanding of how Si effects emerge across the soil–plant–microbiome continuum and helps explain why agronomic responses remain variable and difficult to generalize across contrasting soil and biological systems [28].

In this review, we therefore adopt an integrated soil–plant–microbiome perspective to reassess the role of Si in crop resilience. We first examine the speciation of Si and the controls governing the dissolved, plant-available pool in soil solution. We then analyze how silicate inputs modify soil physical, chemical, and biological functioning, including rhizosphere-associated microbial responses. Next, we discuss plant uptake, transport, and deposition processes and their implications for growth, physiological functioning, and tolerance to abiotic and biotic stresses. Finally, we identify the main scientific and agronomic bottlenecks that currently limit a more rational, predictive, and transferable use of Si in agroecosystems exposed to climate change.

## 2. Silicon Chemistry and Controls of the Dissolved Silicon Pool

The chemistry of Si in soil and plant systems forms the basis of its bioavailability and agronomic functions [29]. Although Si is highly abundant in the Earth’s crust, its availability to plants largely depends on its chemical form and speciation in solution [30]. The dynamics between dissolved, polymerized, and mineral forms of Si, modulated by pH, temperature, ionic interactions, and biological activity, determine the mobility of Si and, ultimately, its accessibility in agroecosystems [26,30,31].

### 2.1. Abundance, Mineral Reservoirs, and Weathering Sources

**Figure 1:**
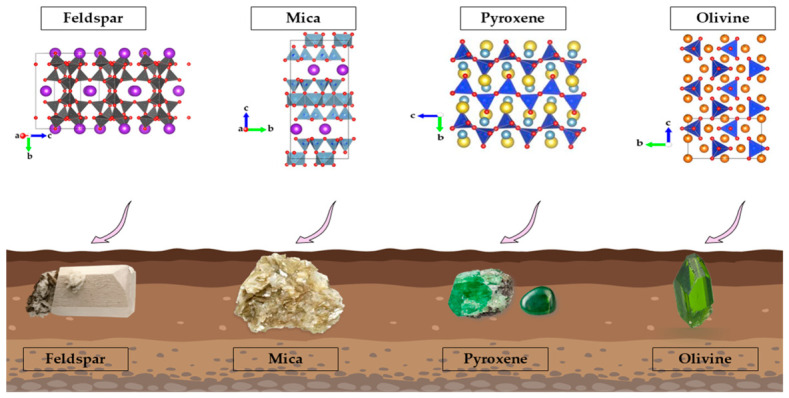
Structures of the main silicate minerals.

Si accounts for about 27.7% of the Earth’s crust, making it the second most abundant element after oxygen [32]. Because of its high affinity for oxygen, Si is found mainly in the form of silica (SiO_2_) and silicate minerals [33]. In soils, these phases represent the major Si reservoirs; however, their bioavailability is controlled less by their abundance than by their reactivity and the rate of weathering. In minimally weathered soils, primary silicates usually dominate the mineral stock and the supply of dissolved Si during dissolution (Figure 1). Feldspars, micas, and other primary silicates are significant sources of Si that are bioavailable due to the process of pedogenesis [30,34]. The reactivity of primary silicates is closely related to their crystalline structure, which is related to the SiO_4_ tetrahedral arrangement in various configurations: isolated tetrahedra, chains, sheets, and three-dimensional frameworks [35].

The alteration of primary silicates results in the formation of secondary minerals, which include clay minerals like kaolinite, smectites, illites, and chlorites that also play a role in the Si balance [36,37]. Nevertheless, the less ordered structures, such as opal, biogenic silica, or some glasses, may have a higher solubility than the crystalline structures. They may also play an important role in the maintenance of Si concentration in the soil solution, despite their lower relative amounts [38,39]. Thus, the agronomic importance of a Si reservoir depends not only on its mass but also on its ability to support, in each soil context, a pool of dissolved Si in the soil solution [30,38]. This distinction between total abundance and effective availability explains why soils rich in Si may nevertheless have limited availability for plants.

### 2.2. Speciation and Dynamics of Dissolved Silicon

In natural systems, Si is dominated by the oxidation state (+IV), which promotes the formation of stable tetravalent compounds, which constitute its main forms in soils [40]. In the mineral phase, it most often adopts tetrahedral coordination, as in most natural silicates [35]. Orthosilicic acid [H_4_SiO_4_ or Si(OH)_4_] is the primary form of dissolved Si under the moderately acidic-to-neutral pH conditions that are characteristic of most agricultural soils [41,42]. As the pH increases, H_4_SiO_4_ gradually deprotonates; the first pK_a_ is close to 9.8, which leads to the formation of H_3_SiO_4_^−^ and increases the propensity of dissolved Si to undergo condensation and polymerization reactions (Equation (1)) [31].

2 H_4_SiO_4_ → H_6_Si_2_O_7_ + H_2_O (1)

When these reactions progress, the equilibrium shifts from the monomer to oligomeric and then polymeric forms, reducing the fraction of H_4_SiO_4_ free, at a high pH and close to saturation [31]. In most agricultural soils, however, concentrations of Si dissolved in soil solution remain low, generally less than one millimolar and often between 0.1 and 0.6 mM [12]. They must therefore be interpreted on the scale of a pool continually replenished by mineral alteration, surface balances, and, where appropriate, silicate inputs [30]. The stability of H_4_SiO_4_ is strongly influenced by the physicochemical environment, with iron (Fe)- and aluminum (Al)-(hydr)oxides surfaces acting as the primary interfaces for its adsorption, while organic matter may compete for these adsorption sites depending on its composition [30,43]. The pH is also an important factor that affects the dissolved Si species as well as the surface charge of the interfaces. Taken together, these controls indicate that Si bioavailability depends on the maintenance of a reactive dissolved pool, whose dynamics are subsequently expressed through the physical, chemical, and biological processes occurring in the soil.

## 3. Soil Responses to Silicon Amendments

**Figure 2:**
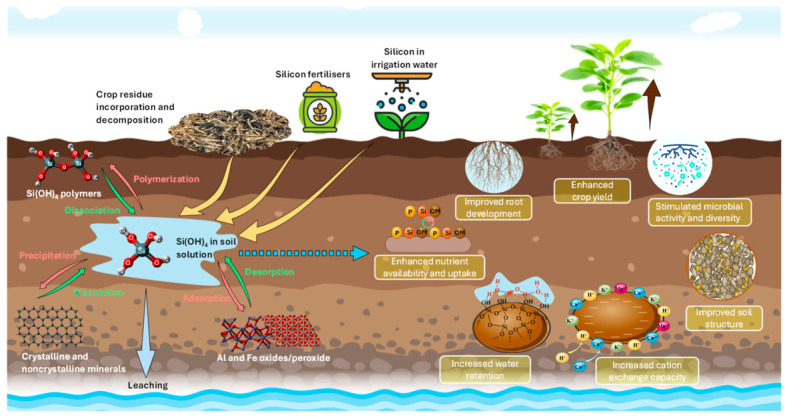
Conceptual framework of silicon functions in the soil–plant–microbiome continuum.

Beyond bringing Si, these amendments modify the functioning of the soil. They influence the structure, the chemistry of the interfaces, and certain biological indicators, with effects that then condition the response of the cultures (Figure 2).

### 3.1. Soil Physical Functioning and Water Relations

#### 3.1.1. Pore Structure, Infiltration, and Water Retention

The use of silicate amendments improves the soil physical properties, directly influencing aeration, infiltration, and water storage capacity [44]. Several studies have reported that silicate amendments reduce bulk density while increasing the total porosity, indicating an improvement in pore structure [45]. However, the magnitude of these effects depends on the type of silicate applied, its application rate, and the soil texture, with more pronounced improvements in bulk density, penetration resistance, and infiltration capacity observed in clay soils [46]. Soil improvements in pore structure have been noted to impact field capacity, which in turn influences yield stabilization, especially under water-limited conditions [47].

#### 3.1.2. Aggregation and Soil Structure Stability

One of the most common physical responses relates to the stability of aggregates, which are critical in the processes of erosion resistance, root penetration, and soil water functionality [48]. Silicate additions can improve aggregate stability through different physico-chemical and biological processes [49]. Si may play a role in binding particles together, particularly through bridging interactions of ions such as Ca^2+^ and Mg^2+^, which are often present in exchangeable forms [49,50]. Studies have shown that there are increases in water-stable aggregates and weighted average diameter, two of the most important properties of aggregate structure [51]. In systems with multiple additions of Si, such as crushed silicated rocks, a cumulative effect was observed, associated with increased hydraulic conductivity and reduced penetration resistance [52]. Si may also have an indirect effect by influencing microbial production of binding agents such as extracellular polymeric substances and compounds of the glomalin type that are critical in aggregate stability at the pore scale [27].

### 3.2. Soil Chemical Functioning

#### 3.2.1. Acidity Regulation and Base Saturation

Ca–Si and Ca–Mg–Si are commonly used as amendments for acidic soils as they increase the number of base cations present and enhance the soil’s buffering capacity [53]. The amendments may increase the soil’s pH by approximately 0.5–2 units, depending on application rate and the soil’s buffering capacity, particularly in highly degraded tropical or subtropical soils [54]. An increase in soil pH is typically accompanied by a reduction in exchangeable acidity and a decrease in Al toxicity at exchange sites, both of which are major constraints in acidic soils [55]. This change also results in a change in the occupation of the exchange sites and a gradual improvement in the status of bases. As the expression of negative charges increases, soils retain more Ca^2+^ and Mg^2+^, and sometimes K^+^, while the effective cation exchange capacity (CEC) may also increase under certain conditions [50]. When silicates are combined with organic amendments, increases in CEC of the order of 30 to 45% have been reported, probably related to an increased expression of the functional loads of organic matter and a better retention of the latter in the soil matrix [56]. In the longer term, the use of Ca-Mg silicates can increase base saturation and modify Ca:Mg:K ratios, with consequences for mineral nutrition [57].

#### 3.2.2. Reactive Surfaces and Sorption Interactions

The chemical effects of silicate amendments extend beyond pH modification and are also expressed via the reactive interfaces, which control adsorption, desorption, complexation, and, in some cases, precipitation or coprecipitation of the solutes [58]. In many soils, Fe and Al oxides and some aluminosilicate border sites are major sorption surfaces for oxyanions [43,58]. Organic matter adds ligands capable of complexing cations and trace metals to this reactivity and can modify the accessibility of mineral surfaces [43]. The silicate amendments can thus modify the mobility of the elements in three main ways: (i) the displacement of the acidity and the state of charge of the surfaces [59]; (ii) competition for certain sorption sites, in particular between oxyanions [60]; and (iii) indirect effects related to the evolution of organic matter when silicates are co-applied with organic amendments [43,58]. This combination explains why the same source of Si can produce contrasting chemical responses depending on the content of Fe/Al oxides, the texture of the soil, and the quality of the organic matter.

#### 3.2.3. Macronutrient Availability

Macronutrient responses arise directly from these changes in pH, surface charge, and interfacial reactivity. In the case of phosphorus, which is typically strongly retained on hydroxylated surfaces of Fe/Al (hydr)oxides, increases in dissolved P are primarily governed by two mechanisms: (i) reduced fixation under decreasing acidity, and (ii) sorption competition, which may displace a fraction of previously sorbed phosphate or reduce the effective sorption capacity [60]. However, the magnitude of the effect depends on the initial pH, the abundance of oxides, and the timing of applications [61]. For nitrogen, the effects of Si are most often indirect. Reduced acidity and Al^3+^ stress can promote microbial processes such as mineralization and nitrification, thereby increasing the net availability of nitrogen [44,53,62]. In acidic soils, this stimulation may improve nitrate supply but increase the risk of leaching if synchronization with plant uptake is insufficient, particularly in coarse soils [62]. In leguminous plants, silicon has been reported to promote nodulation and improve symbiotic N_2_ fixation in certain systems, although the magnitude of these effects remains context-dependent [63,64]. For potassium (K), the response often combines alteration of the exchange equilibria with a direct fertilizing effect when the applied source contains K [59,65]. More favorable exchange chemistry can improve the retention of K^+^ between the soil solution and the exchangeable or partially fixed phases, while K-silicates act simultaneously as a source of H_4_SiO_4_ and K [59,66]. Therefore, it is essential to distinguish the specific effect of Si from the fertilizing effect associated with the applied formulation [66].

#### 3.2.4. Micronutrients and Potentially Toxic Elements

The responses of Fe and Manganese demonstrate a greater degree of context dependence; in certain acidic soils, reduced solubility may mitigate toxicities, while in other systems, it may limit the effectiveness of their availability, depending on soil speciation and the redox state [67,68]. In general, silicate amendments are linked to a reduction in bioavailability of potentially toxic metals [69]. This is mainly due to adsorption, complexation, precipitation, and coprecipitation processes occurring at reactive interfaces [69,70]. By reducing the dissolved and labile pools in the rhizosphere, the uptake by the plant and the exposure to microorganisms are altered [71].

### 3.3. Soil Biological Responses: Enzymatic Activity and Microbial Dynamics

Soil’s enzymatic activities and the structure of microbial communities represent sensitive bioindicators of the biological activity of soils and the corresponding reaction to management practices [27]. These bioindicators are used to describe the cumulative effects of soil amendments on micro-environments, resource availability, and biotic interactions [72,73]. The literature does not confirm the presence of a universal and unequivocal link between the silicate amendments and the biological response; rather, it indicates the presence of trends with a variable degree depending on the formulation and the organic components added along with the soil.

#### 3.3.1. Enzymatic Responses as Functional Indicators

These enzymatic reactions to Si amendments are often used as an indicator of a shift in the microbial management of substrates and their functional priorities. Several studies have demonstrated an increase in extracellular enzymes associated with the depolymerization of plant materials, particularly those of β-glucosidase and cellulase, during the simultaneous addition of Si and organic resources [74,75]. This may reflect enhanced processing of labile organic materials and/or improved microbial access to substrates. In contrast, the responses of oxidative enzymes, typically associated with the transformation of more recalcitrant materials, are more variable, suggesting tighter regulation by substrate quality and the functional composition of the microorganism’s community, particularly fungi [76]. There have also been studies indicating changes in the amount of acquisition enzymes such as urease and phosphatases [27,77], but these responses must be considered carefully because they are more functional adjustments of the microorganisms than actual increases in nutrient availability.

#### 3.3.2. Microbiome Structure and Network Organization

Advances in high-throughput sequencing have shown that silicate amendments can influence the composition of microbial communities in both the rhizosphere and bulk soil. However, the magnitude and direction of these responses are highly context-dependent, varying with soil type, amendment characteristics, organic co-inputs, crop species, and prevailing stress conditions [27,78]. Rather than reflecting a direct effect of Si alone, these shifts are more accurately attributed to the broader reconfiguration of rhizosphere conditions induced by silicate inputs, including changes in pH, exchange equilibria, reactive mineral surfaces, nutrient availability, metal mobility, soil aggregation, and microhabitat heterogeneity [27,53]. Simultaneously, the improvement of plant performance under Si supply may alter root growth and rhizodeposition patterns, which further contributes to reshaping the microbial population and selection in the rhizosphere.

At the bacterial level, prevailing trends indicate an enrichment of taxa associated with rapid substrate renewal, nutrient transformations, and rhizosphere responsiveness, especially within Proteobacteria and Actinobacteria. In contrast, Acidobacteria exhibit more variable responses based on edaphic conditions and resource availability [27,78]. Responses within fungal communities exhibit variability, potentially resulting in alterations in dominant saprotrophic groups like Ascomycota and Basidiomycota. Conversely, the response of arbuscular mycorrhizal fungi seems contingent upon host identity, soil limitations, and the equilibrium between the nutritional and structural impacts of the amendment [75,79].

Functionally, these patterns indicate that silicate amendments do not promote a generalized “Si microbiome,” but instead alter environment-specific filters that regulate microbial selection and activity. Recent field studies further support this context-dependent interpretation. In two years of maize research, the introduction of a synthetic consortium of silicate-solubilizing bacteria markedly modified rhizosphere microecology. This treatment also increased plant Si concentration and the expression of related transporters. Moreover, it was linked to changes in several bacterial genera that are known to affect plant nutrition [80]. Importantly, microbial diversity and network structure did not respond in a uniformly positive manner, indicating that microbiome reconfiguration under Si-related treatments should be interpreted as selective and system-dependent rather than uniformly beneficial [80]. The study reports changes in the rhizosphere microbial taxonomic landscape under SSB SynCom application and identifies genus-level shifts alongside co-occurrence network responses, while also linking these changes to improved Si acquisition and plant performance.

From a mechanistic perspective, a key observation is that silicate amendments can restructure microbial communities by modifying the physicochemical framework that governs microbial selection. Enhanced aggregation and pore connectivity may influence oxygen transport, water retention, and substrate accessibility at the microscale, while alterations in nutrient retention and metal speciation can vary the competitive dynamics for microbes [27,53]. Moreover, microorganisms participating in silicate weathering may interact at mineral interfaces by adhering to surfaces, forming biofilms, releasing organic acids, chelating, and inducing localized alterations in pH or redox conditions, consequently altering the availability of mineral-associated nutrients. The recent study endorses the function of silica-solubilizing rhizobacteria as bioinoculants for sustainable silica management, emphasizing genera such as *Bacillus*, *Pseudomonas*, and *Burkholderia* as significant silica-solubilizing groups [55,81]. Meanwhile, Timmermann et al. [55] demonstrate that *B. subtilis* augmented silicate dissolution via biofilm-associated and pH-mediated mechanisms. Plant-mediated feedback is also likely to be important, by affecting root architecture, stress intensity, and tissue nutritional status. Si may indirectly change the quantity and composition of rhizodeposits, which are major drivers of microbiome assembly [27,53]. Accordingly, the observed microbial shifts should be interpreted as components of a broader soil–plant–microbiome reorganization rather than as isolated taxonomic responses. Co-occurrence network analysis has shown, in some systems, alterations in microbial connectivity due to silicate amendment or inoculation protocols [27,80]. Nevertheless, these findings must be interpreted with caution. Network metrics obtained from sequencing data generally indicate patterns of statistical correlation and do not, on their own, provide evidence of direct ecological interaction, functional complementarity, or enhanced stability of the soil microbiome. Their interpretation is also affected by sample design, sequencing depth, taxonomic resolution, and bioinformatic processing.

The available literature supports the view that Si amendments can reshape soil microbial communities and associated functional proxies, but the causal chain linking microbiome reorganization to enhanced Si mobilization, greater plant Si uptake, or improved crop performance remains insufficiently resolved (Table 1). These observations indicate that silicate amendments, beyond their role in plant Si supply, also function as modifiers of the soil environment, and that their agronomic value will depend on their capacity to enhance plant acquisition and transport of Si to the tissues, which is addressed in the next section.

## 4. Plant Acquisition of Silicon: Flux Constraints, Transport, and Deposition

The agronomic effects of Si depend on the plant’s ability to intercept dissolved Si, transport it through root tissues, and redistribute it to the organs where it is deposited. This process constitutes the direct link between the availability of in the rhizosphere solutions and its accumulation in plant tissue.

### 4.1. Dissolved Silicon Supply as a Flux Constraint

In agricultural soils, dissolved Si concentrations are generally low (typically sub-millimolar), meaning that even highly accumulating species such as rice grow in a diluted environment and must efficiently acquire and utilize Si from a limited external supply [92]. The bioavailability of Si is therefore interpreted above all as a flow to the roots. When the conditions approach saturation, the condensation of dissolved Si may be limited by kinetic constraints, allowing a mobile fraction to be maintained and remain available for plant uptake [93]. In practice, Si uptake depends on the ability of the soil to maintain a continuous supply of the dissolved Si in the rhizosphere [30].

### 4.2. Root Uptake, Radial Transport, and Shoots Deposition

The entry of Si into the plant follows the general organization of root acquisition, with a particular constraint linked to the generally low external gradient. A significant routing to the stele therefore requires specialized transport components [94]. In rice, absorption is based on the coordinated action of the Lsi1 inlet channel, an NIP-type aquaporin, and the efflux transporter Lsi2, which together ensure the radial transfer of the Si to the xylem [95,96]. Their polarized location in the exoderm and endoderm provides directionality to the transport to the xylem and partly explains the strong accumulation capacity of this species (Table S1). In rice, a significant proportion of the variability of Si accumulation among cultivars is explained by differences in abundance, activity, and regulation of these transporters, directly linking phenotypic diversity to a defined mechanistic base [97]. Functional counterparts have been identified in other species; however, the level of mechanistic understanding remains uneven, and generalization of established models in rice should be approached with caution [95].

**Figure 3:**
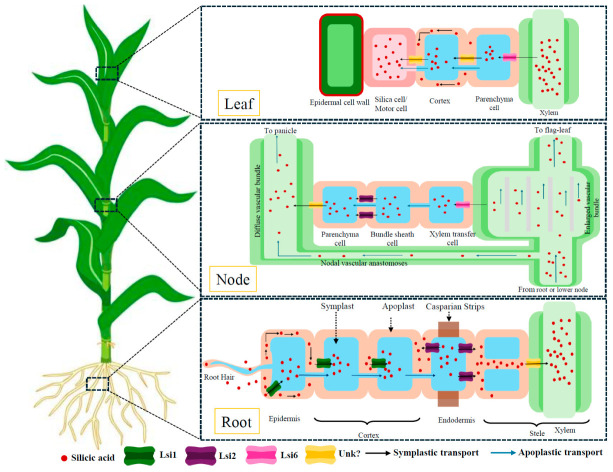
Schematic representation of silicon forms in soil, root uptake via Lsi transporters, long-distance transport and tissue-specific deposition in plants. Adapted from [99].

Once loaded into the xylem, Si is transloaded to the transpiring organs, where it accumulates and precipitates when local concentrations exceed solubility limits, leading to the formation of amorphous silica (SiO_2_·nH_2_O) and phytoliths [93]. In rice, Lsi6 participates in tissue redistribution and may promote the allocation of Si to certain aerial and reproductive organs [94]. Si deposition in epidermal, vascular, or supportive tissues can organize into structured architectures, notably in the form of a cuticle–silica layer or phytoliths, which reinforce cell walls and increase tissue rigidity [10,98]. As the plants senesce, phytoliths accumulate in the plant residue, releasing Si back into the soil after plant decay [98]. The agronomic significance of Si acquisition depends on the coupling between dissolved Si supply in the rhizosphere and the transport machinery governing its radial transfer, xylem loading, and tissue redistribution (Figure 3). This interface between soil availability and plant transport capacity largely determines the extent to which Si-mediated functions can be expressed in plant growth, development, and physiology, as discussed in the next section.

## 5. Physiological Roles of Silicon in Plant Growth and Development

After assimilation and deposition in tissues, Si affects plant performance by influencing organ mechanical strength, canopy architecture, and photosynthetic activity.

### 5.1. Mechanical Reinforcement and Lodging Resistance

High Si accumulation enhances the mechanical strength of aboveground plant organs through the deposition of silica within cell walls and epidermal tissues [100]. In rice crops, substantial Si accumulation in aerial parts reinforces its role as a key structural component, particularly in maintaining culm rigidity [101]. One of the most extensively documented effects of Si in rice is the mitigation of lodging, a major factor in grain yield loss due to reduced grain filling, reduced harvesting efficiency, and potentially increased disease risk [102,103]. By increasing the resistance of stems and tissues, Si improves the mechanical strength of the canopy, especially in dense or intensive fertilization.

### 5.2. Root and Shoot Development and Yield Components

Si application has demonstrated positive associations with growth and yield, particularly under conditions of low Si availability or environmental constraints [104]. Numerous studies have reported that Si enhances root system development, including via increases in root length, branching, and biomass, thereby improving soil exploration for water and nutrient acquisition [105,106]. At the shoot level, Si contributes to increased leaf area and delayed senescence, extending the functional lifespan of the canopy during critical reproductive growth [107,108]. These combined effects can improve grain filling efficiency and increase grain weight [107,109].

### 5.3. Photosynthetic Performance and Canopy Functioning

**Figure 4:**
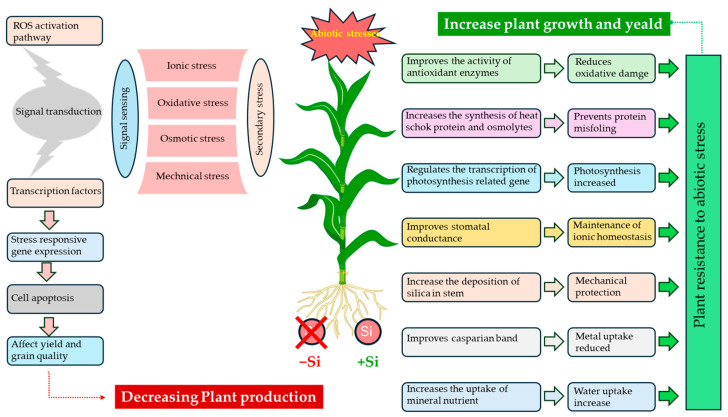
Physiological effects of silicon on plant growth and development. Adapted from [113].

Si influences photosynthesis performance primarily through its effects on canopy structure and preserving the functional integrity of leaves. By promoting a more erect leaf architecture, Si reduces self-shading within the canopy and improves radiation penetration, promoting more uniform light use in dense stands [54]. At the foliar level, Si plays a role in the better conservation of chlorophylls and carotenoids [110], and in the better conservation of photosystem II (PSII) fluorescence indicators (Fv/Fm; ΦPSII), particularly under stress conditions [111]. Modulations in gene expression related to photosynthesis (PetE and Psb proteins) have been noted, with varying levels of importance depending on the species and conditions [112]. These observations suggest that the effects of Si on plant growth and development result from coordinated structural and physiological adjustments rather than a single dominant mechanism (Figure 4). Their agronomic is particularly relevance is particularly evident under conditions of intense and/or recurrent stress. This underpins the mechanisms of Si-mediated tolerance to abiotic stress tolerance discussed in the following section.

## 6. Silicon-Mediated Mechanisms of Abiotic Stress Tolerance

The effect of silicon on abiotic stress tolerance is governed by four closely interconnected processes: (i) improvement of water relations and osmotic adjustment, (ii) maintenance of ionic balance, (iii) restriction of the uptake and translocation of metals or metalloids, and (iv) maintenance of redox homeostasis. These processes are not mutually exclusive; rather, improvements in water relations or ionic balance help limit secondary damage and supports plant performance.

### 6.1. Drought and Osmotic Stress: Water Status and Hydraulics

Under drought and osmotic stress conditions, Si supplementation contributes to improved plant performance by enhancing water acquisition, reducing non-productive water losses, and promoting more efficient osmotic adjustment [114,115]. Several studies report that under water deficit, an improvement in root architecture length, branching, or biomass favors the exploration of larger volumes of soil and supports the water supply when the surface layer dries [116]. However, these responses remain variable depending on the genotype, the intensity of stress, and the initial status of the soil. In addition, silicon is related to lower rates of basal water loss and to the maintenance of stomatal function, which is beneficial to carbon assimilation that is correlated to thermoregulation processes [10,117]. It has been reported that silicon is associated with increased root hydraulic conductance, often accompanied by aquaporins, particularly PIP subtypes, as demonstrated by Liu et al. [118], and Saja-Garbarz et al. [115]. At the cellular level, Si is also associated with an increased accumulation of compatible solutes (e.g., proline, glycine betaine, and soluble sugars), contributing to the maintenance of turgor and the protection of macromolecules under dehydration conditions [119]. This combination results in higher relative water content and better preservation of leaf function under drought conditions [71,120].

### 6.2. Salinity: Ion Homeostasis and Barrier Functions

The alleviation of salt stress by Si is primarily attributed to the synergistic effects of the improvement of water retention capacity and the regulation of ionic homeostasis, especially the maintenance of the K^+^/Na^+^ ratio [114,121]. Si application is commonly associated with reduced accumulation of Na^+^ and in some cases Cl^−^ in the aerial tissues [122]. As reported by Heidarpour et al. [123], Si-mediated tolerance involves multiple mechanisms, including reduced Na^+^ uptake, enhanced retention of Na^+^ in the root tissues, and limited translocation to the shoot. Simultaneously, Si is frequently linked to enhanced K^+^ status and the stabilization of the K^+^/Na^+^ ratio, thereby promoting enzymatic and metabolic stability under saline conditions [124,125]. These effects are frequently associated with the modulation of ion transporters systems, including the activation of K^+^ uptake or retention pathways and the restriction of specific Na^+^ entry pathways, particularly via non-selective cation channels [126]. In addition, Si deposition in root tissues may reduce, bypass flow and limit passive salt entry into the stele through a barrier effect [127].

### 6.3. Metals and Metalloids: Rhizosphere Immobilization and Internal Sequestration

The mitigation of metals and metalloids toxicity by Si involves a continuum of mechanisms, the relative importance of which depends on element speciation, mobility in the rhizosphere, and the ability of the plant to limit their uptake and internal redistribution. In most cases, three main levels of action can be distinguished: (i) reduction in bioavailability in the rhizosphere through adsorption, complexation, precipitation, or coprecipitation; (ii) restriction of root absorption and root–shoot translocation via the reinforcement of apoplastic barriers and the modulation of transporters systems; and (iii) increased immobilization within plant tissues through cell wall binding and/or vacuolar sequestration [128,129,130]. For certain metalloids, speciation is decisive role. In flooded systems, arsenite [As(OH)_3_, As(III)] shares with H_4_SiO_4_ a neutral form at physiological pH, which allows it to use certain transport routes associated with Si [131]. Under these conditions, Si addition can reduce arsenic accumulation by limiting its entry by competition at the root interface and, in certain contexts, by modulating the expression or the activity of components involved in the transport of Si [131,132]. However, responses observed in rice strongly depend on the flooding regime, the redox control of the rhizosphere, and the availability of Fe oxides, which influence the bioavailable fraction of arsenic [131,133,134]. In non-flooded systems, Cd is a central case. Si-mediated attenuation often results in decreased labile fractions in the soil, increased immobilization in the root, and limited translocation to shoots and harvesting organs [32]. In the plant, this response frequently involves enhanced binding to the parietal fractions (e.g., pectins and hemicelluloses) as well as vacuolar sequestration associated with ligands such as phytochelatins [135]. Similar trends have been reported for other metals (e.g., Pb and Cr), with increased retention in root and restricted translocation to aerial parts [136,137,138]. Reduced exposure to toxic elements may also contribute to improvements in nutritional status of N, P, or K, not as a direct effect of Si, but as a consequence of preserved root function and reduced toxic interference [139]. The evidence for a protective role of Si against metals is strong, but the dominant mechanism varies by element, speciation, soil, and plant species (Table S2).

### 6.4. Redox Homeostasis: Antioxidant Systems and Signal Regulation

**Figure 5:**
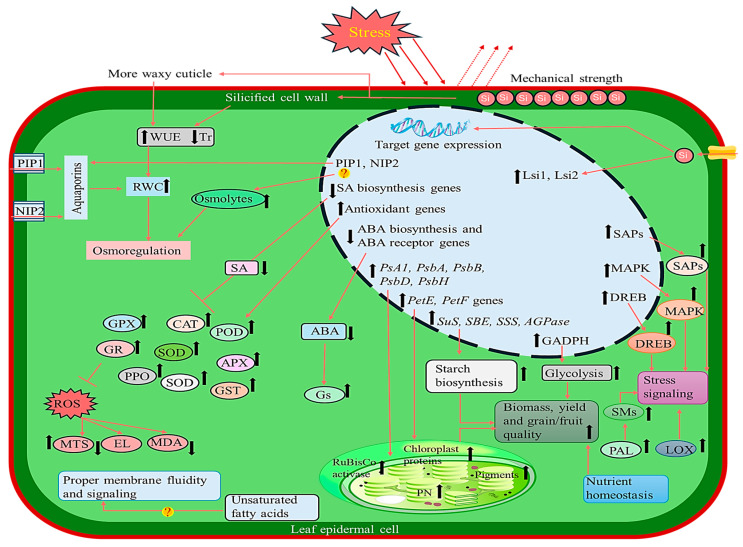
Cellular and molecular basis of silicon-mediated plant performance and stress adaptation (↑ increase; ↓ decrease). Adapted from [104].

Under abiotic stress, Si is frequently associated with improved redox homeostasis in plants [140]. Drought, salinity, and metal toxicity enhance the production of reactive oxygen species (ROS) and, often, reactive nitrogen species (RNS), which can damage membranes, proteins, and nucleic acids when their accumulation exceeds cellular detoxification and repair capacity [140,141]. At the enzymatic level, Si is often associated with increased activities of superoxide dismutase, catalase, peroxidases, and enzymes involved in the ascorbate–glutathione cycle [140]. Several studies have also reported better maintenance of ascorbate and glutathione pools, along with more favorable ratios of reduced to oxidized ascorbate (AsA/DHA) and reduced to oxidized glutathione (GSH/GSSG) [140,142]. Functionally, these adjustments are often accompanied by lower levels of oxidative damage markers, particularly malondialdehyde, indicating reduced lipid peroxidation rather than its induction [143,144,145]. In addition, Si may also contribute to the modulation of stress signaling, although the underlying mechanisms remain only partially understood [112]. Si may act not only through antioxidant reinforcement, but also by alleviating upstream stress constraints that promote ROS/RNS generation [115]. The current evidence, therefore, supports a multi-layered mode of action, integrating indirect effects on stress intensity with more direct influences on defense and signaling pathways [146]. Together, these mechanisms indicate that Si improves tolerance to abiotic stresses by acting on water relations, ionic homeostasis, limitation of exposure to toxic elements, and redox homeostasis (Figure 5). this integrated mode is not restricted to abiotic stress but can be extended to plant interactions with pathogens and pests through structural and inducible mechanisms that enhance host resistance, as discussed in the next section.

## 7. Silicon-Mediated Mechanisms of Biotic Stress Resistance

Si-mediated resistance to biotic stress is based on two complementary components: (i) constitutive reinforcement of physical barriers, which increases the invasion threshold and reduces the feeding efficiency of certain pests; (ii) an inducible component involving the activation of immune and metabolic responses, allowing faster or more intense responses upon attack. The agronomic relevance of this dual mechanism lies in the ability of Si to influence both host exposure and the effectiveness of its defense response.

### 7.1. Priming and Induced Defenses: Jasmonic Acid, Salicylic Acid, and Phenylpropanoid Pathways

Beyond the structural effects, Si is frequently associated with a priming of the defenses, allowing a faster or more intense activation after attack [147]. In several systems, the jasmonate pathway appears as a recurring node: Si can amplify attack-induced accumulation of jasmonic acid (JA) and enhance the expression of defense genes associated with this pathway, contributing to resistance against certain herbivorous insects and, in some cases, necrotrophic pathogens [148]. Si is also associated with an increase in enzymatic activities such as peroxidase, polyphenol oxidase, and phenylalanine ammonia lyase, consistent with the activation of the phenylpropanoid pathway, parietal strengthening, and increased accumulation of defense metabolites [149]. The intensity of these responses, however, depends on the system studied and the attack conditions. An implication of the salicylate pathway (SA) is suggested in several cases, but the mechanistic resolution often remains less clear than for JA and deserves a cautious interpretation [150]. Si can finally modulate herbivore-induced volatile organic compounds, paving the way for indirect defense via the attraction of natural enemies [151]. However, the functional scope of this modulation depends on the ecological context and must be validated on a case-by-case basis.

### 7.2. Fungal Diseases: Rice Blast as a Model System

Rice blast (*Magnaporthe oryzae*) is one of the best-documented systems to illustrate Si-mediated fungal resistance. Several studies show an earlier or more intense activation of defense genes, in particular pathogenesis-related proteins such as chitinases, β-1,3-glucanases, and thaumatin-type proteins [152]. Si is also associated with an increase in diterpenoid phytoalexins (e.g., momilactones, phytocassanes, and oryzalexins) that may limit fungal growth [153]. This system illustrates a case where the activation of the inducible defenses and the tissue reinforcement converge in a particularly clear manner, without constituting a universal model. Synergies between mineral nutrition and plant defense have also been recorded. For example, the combination of K + Si or zinc and Si (Zn + Si) has been shown to have a greater effect on reducing disease severity than the individual elements [154]. This evidence suggests that there is an additive effect between nutrition, fortification, and immunity. Nano-formulations of silica shown also promising results, but their agronomic value must be evaluated in a comparative manner (e.g., active dose, persistence, and environmental fate) under field conditions [155].

### 7.3. Herbivores and Mites: Feeding Deterrence and Indirect Defense

**Figure 6:**
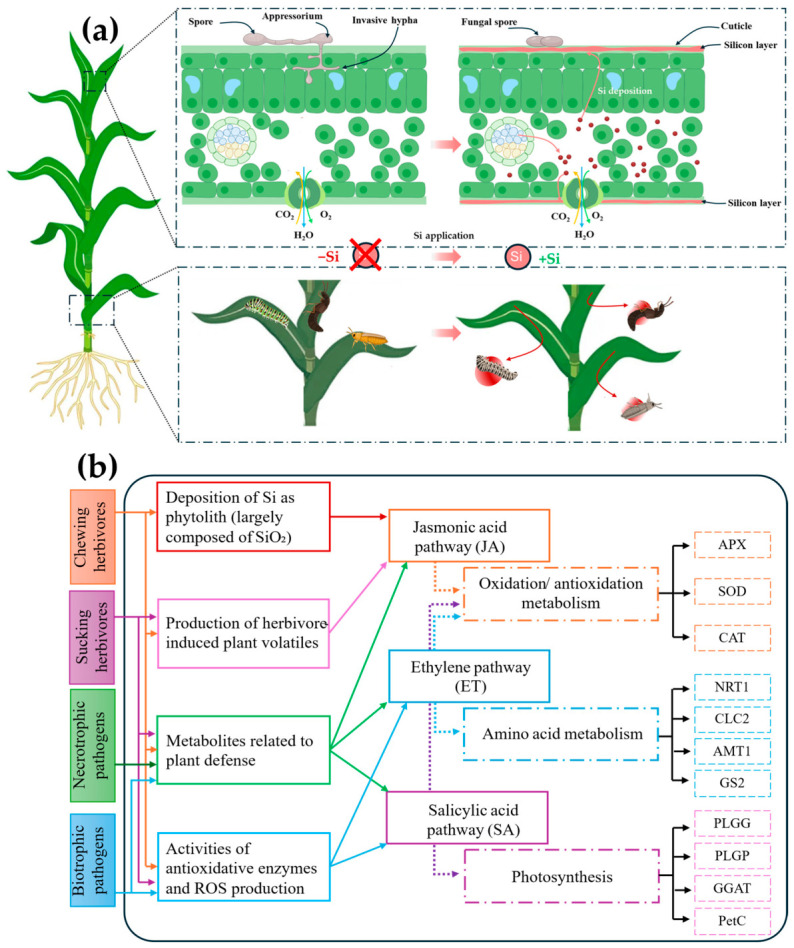
Integrated mechanisms of silicon-mediated defense against biotic stresses: (**a**) Structural and inducible defenses in plants; (**b**) defense signaling pathways involved in responses to herbivores and pathogens. Adapted from [161,162].

Si reduces the damage caused by many pests by combining mechanical effects and inducible responses. Silicification of plant tissues makes leaves harder and more abrasive, which could hinder the feeding of chewing insects [156]. Consequently, this could lead to less effective feeding and possible harm to their mouthparts [157]. In piercing–sucking pests, it can hinder stylet penetration and reduce access to the phloem [158]. These processes are accompanied by an increase in enzymatic and phenolic defensive reactions after infestation. This is consistent with the strengthening of induced defense responses, as proposed by Qi et al. [149] and Kobyletska et al. [159]. However, the effects of Si on pests are not uniform: they generally appear to be more pronounced in external herbivores than in organisms that develop inside tissues, which are able to partially bypass mechanical barriers or locally modify the organization of attacked tissues [160]. Similarly, Si most often acts as a factor that enhances resistance rather than as a substitute for genetic determinants or other components of integrated protection. The available data supports a model in which Si enhances resistance to biotic stress through a combination of structural effects, the priming of defenses, and, in some systems, favorable ecological interactions (Figure 6). However, the magnitude of these effects remains variable depending on the pathosystem and the level of Si accumulation, which reinforces the need for causal validation and field tests, as discussed in the following section.

## 8. From Mechanisms to Field Applications: Constraints and Research Priorities

Despite growing evidence supporting the agronomic relevance of Si within the soil–plant–microbiome continuum, several bottlenecks still limit their translation into robust, generalizable, and economically rational recommendations. Future progress will depend less on the accumulation of additional descriptive examples than on the ability to prioritize mechanisms, standardize experimental protocols, and identify conditions under which Si responses become truly predictable. The first major challenge concerns the clarification of causal mechanisms. Many Si-associated responses are well documented at the phenotypic and physiological levels, yet often remains unresolved whether these responses result from direct Si effects or from indirect effects is mediated through changes in pH, nutrition, soil structure, or rhizospheric functioning. This ambiguity is particularly important for redox homeostasis, hormonal interactions, and some aspects of priming. A key priority is therefore to better connect Si transport, deposition, signaling, and functional outcomes, while distinguishing effects directly attributable to Si from those arising from broader alleviation of stress constraints. A second major bottleneck concerns the interactions between Si and the microbiome.

The available data reveal convincing associations among Si inputs, enzymatic activities, and microbial community reorganization, but causal evidence linking these shifts to effective Si mobilization or improved plant performance remains limited. Future research will need to move beyond predominantly associative approaches by incorporating stronger functional validations based on measurable microbial traits, targeted manipulations, and experimental designs that explicitly test feedback among plants, the rhizosphere, and the Si cycle. A third challenge relates to the diversity of Si sources and formulations. Soluble, stabilized, and solid sources differ markedly in their release kinetics, effects on soil properties, and suitability across soil contexts. Research priorities therefore lie not only in developing new formulations, but also in positioning them rationally according to soil type, initial Si status, cropping system, and the nature of the dominant constraints.

Controlled-release strategies and some nano-formulations appear promising, but their actual value must be assessed through rigorous comparisons integrating active dose, persistence, environmental fate, and net agronomic benefit. A fourth bottleneck concerns the inter- and intra-specific variability of Si acquisition and use. In highly accumulative species, several transport mechanisms are already well characterized, particularly in rice. By contrast, the genetic and functional basis of Si-use efficiency remains unevenly documented in many other crops. Quantitative genetics, genome-wide association approaches, and, potentially, genome editing may help define ideotypes integrating uptake capacity, tissue allocation, and stress performance. However, these strategies should be accompanied by a systematic assessment of potential trade-offs involving growth, yield, and quality. Finally, scaling remains the decisive test of the agronomic value of Si. Multi-site and multi-season trials covering gradients of soils, climates, genotypes, and stress intensities are essential to determine under which conditions Si responses are most reproducible. Such trials should assess not only yield, but also production stability, product quality, and, where relevant, economic profitability. They should also be coupled with improved diagnostic tools for plant-available Si response indicators, to move beyond generic application strategies toward more targeted and predictive management. These priorities indicate that the challenge is no longer simply to document beneficial Si effects, but to define the conditions under which they become mechanistically interpretable, agronomically reproducible, and transferable into field practice.

## 9. Conclusions

Silicon should be viewed not as an isolated input, but as a component of the soil–plant–microbiome continuum whose agronomic effects emerge from interactions among soil chemistry, rhizosphere functioning, plant acquisition, and stress physiology. Across the literature, the most consistent finding is that Si enhances crop resilience to multiple abiotic and biotic stresses; however, these benefits are highly context-dependent. Their magnitude varies with the initial silicic status of the soil, the source and formulation applied, the mode of application, plant genotype, and the nature, duration, and intensity of the prevailing stress. At the same time, this synthesis highlights several major limitations in the current state of knowledge. First, many of the reported effects of Si remain difficult to disentangle because it is often unclear whether they result from direct Si-mediated mechanisms or from indirect changes in pH, nutrient availability, soil structure, or plant vigor. Second, although growing evidence suggests that Si-related inputs can reshape rhizosphere microbial communities, causal links between microbiome reorganization, enhanced Si mobilization, and improved plant performance remain insufficiently demonstrated. Third, the diversity of Si sources, formulations, and application strategies continues to outpace the development of standardized comparative frameworks, which limits the transferability of results across studies and agroecosystems.

Future research should therefore move toward more integrative and predictive approaches. Priorities include multi-scale studies linking Si speciation, soil processes, microbial traits, plant transport, and stress responses; function-oriented experiments capable of distinguishing correlation from causation in the rhizosphere; better evaluation of genotype × soil × formulation interactions; and multi-site, multi-season trials assessing not only yield response, but also response stability, quality, and economic relevance. Progress in these areas will be essential to transform a large but still fragmented body of evidence into transferable agronomic rules.

In this perspective, the key challenge is no longer simply to show that Si can be beneficial, but to determine under which conditions it becomes a reliable and predictable lever for crop management. A more mechanistic and continuum-based understanding of Si across the soil–plant–microbiome system will therefore be central to its rational integration into climate-resilient and resource-efficient agriculture.

## Figures and Tables

**Table 1:** Reported effects of silicon amendments on soil physical, chemical and biological properties and agronomic implications.

Domain	Main Effects of Si Amendments	Main Processes Involved	Agronomic Relevance	References
Physical functioning	Improved aggregation, porosity, infiltration, and water retention	Particle binding, improved aggregate stability, and changes in pore organization	Better root penetration, aeration, and water availability	[82,83]
Acidity and exchange chemistry	Increased pH and base saturation; reduced exchangeable acidity and Al stress	Release of base cations, buffering, and Al–Si interactions	Improved root environment and nutrient availability in acidic soils	[84,85]
Nutrient availability	Increased plant-available Si and, in some contexts, improved N, P, and K availability	Silicate dissolution, sorption competition, and rhizosphere effects	Better crop nutrition and greater stress resilience	[86,87]
Potentially toxic elements	Reduced bioavailability and plant uptake of toxic metals	Adsorption, complexation, precipitation/coprecipitation, and restricted translocation	Lower phytotoxicity and safer crop production in contaminated soils	[69,88]
Microbial and enzymatic activity	Increased microbial biomass and shifts in enzyme activities linked to nutrient cycling	Improved microhabitats, substrate availability, and rhizosphere activity	Enhanced soil biological functioning and nutrient transformation	[74,89]
Microbiome structure	Context-dependent changes in bacterial and fungal community composition	Rhizosphere reconfiguration driven by pH, nutrient status, aggregation, and plant feedback	Potential improvements in rhizosphere functioning, although causal links remain incomplete	[27,80]
Plant health and stress-related outcomes	Lower disease incidence and improved tolerance to abiotic and biotic stresses	Structural reinforcement, defense priming, and improved physiological regulation	Greater yield stability and reduced input dependence under stress	[90,91]

## Data Availability

No new data were created or analyzed in this study. Data sharing is not applicable to this article.

## Supplementary Materials

The following supporting information can be downloaded at: https://www.mdpi.com/article/10.3390/plants15091320/s1. Table S1: Silicon transporters identified in higher plants, their localization and functional roles in silicon uptake and distribution; Table S2: Main mechanisms by which Si mitigates the toxicity of selected metal(loid)s in soil–plant systems. References [162,163,164,165,166,167,168,169,170,171,172,173,174,175,176,177,178,179,180,181,182,183,184,185,186,187,188,189,190,191,192,193] are cited in the supplementary materials.
