# Supplementary material for: Silicon at the Soil–Plant–Microbiome Interface: Rhizospheric Reconfiguration and Crop Resilience to Environmental Stresses
Source: Plants (Basel). 2026 Apr 25;15(9):1320. doi: 10.3390/plants15091320 (PMC13165089; doi:10.3390/plants15091320)
Supplement: Supplementary file 1 [file plants-15-01320-s001.zip › plants-4222425-supplementary.pdf]

Supplementary Information

Table S1. Silicon transporters identified in higher plants, their localization and functional roles in silicon uptake and distribution.

Gene name	Protein family	Transport direction	Tissue Localization	Subcellular localization	Functional roles	Knockout phenotypes	Overexpression phenotypes	Selected orthologs & plant species	Functional characteristics	Reference
OsLsi1 HvLsi1 ZmLsi1 SlLsi1	NIP;NIP-III	Influx $Si(OH)_4$ uptake; bidirectional for As(III) & Sb(III)	Root tips, basal root zones, xylem parenchyma (shoots), leaf blades & sheaths	Plasma membrane; polar at lateral domain (exodermis/endodermis); non-polar in root tips; polar toward xylem vessels in xylem parenchyma	Primary Si influx transporter; root-to-xylem Si loading; xylem unloading in shoots; Si distribution to developing organs; As(III), Sb(III) uptake	Reduced Si in roots/shoots; decreased As/Sb accumulation; reduced Si in cell sap; increased tolerance to As/Sb toxicity; impaired Si transport	AtLsi1 overexpression increases Si in roots; SlLsi1 overexpression increases Si in root cell sap but not in shoots	Rice (*OsLsi1*/*OsNIP2;1*), Barley (*HvLsi1*), Maize (*ZmLsi1*), Tomato (*SlLsi1*), Cucumber (*CsLsi1*), Finger millet (*EcLsi1*), Soybean (*GmNIP2.1*), Ryegrass (*LpLsi1*)	Two NPA motifs; GSGR/STAR selectivity filter; bidirectional permeability; constitutive expression; CME-independent polar localization	[163–165]
OsLsi2 HvLsi2 ZmLsi2 SlLsi2	Anion/H^+ antiporter; FAR family; ArsB-like transporter	Efflux ($Si(OH)_4$ loading into xylem); pH-dependent	Root endodermis & exodermis, node I transfer cells, xylem parenchyma	Plasma membrane; polar at lateral domain (endodermis), overlap at the transversal side of the exodermis; opposite polarity to Lsi1 at nodes	Si efflux transporter; active Si loading into xylem; radial Si transport; As(III), Sb(III) efflux; intervascular Si transfer at nodes	Reduced Si in xylem sap; no effect on root Si; impaired xylem loading; reduced aerial tissue Si; increased As/Sb accumulation in endodermal vacuoles	*CsLsi2* expression in tomato increases Si uptake & shoot Si accumulation; enhanced drought & heat tolerance	Rice (*OsLsi2*), Barley (*HvLsi2*), Maize (*ZmLsi2*), Tomato (*SlLsi2*), Cucumber (*CsLsi2*), Finger millet (*EcLsi2*), Soybean (*GmLsi2-like*)	H^+/Si antiporter; ancient gene; pH-dependent activity; constitutive expression; characterized by polarity opposite to Lsi1	[95,166]
OsLsi6 HvLsi6 ZmLsi6 EcLsi6	NIP; NIP-III	Influx; xylem unloading of Si; intervascular transfer	Root epidermis, cortex, root tips; leaf blade/sheath vascular bundles; node I transfer cells; panicles	Plasma membrane; polar toward transfer cells at node I (proximal side); non-polar in root tips	Si influx at root tips; xylem unloading of Si in leaves; intervascular Si transfer in nodes; maintains Si distribution gradient; panicle Si accumulation	Reduced Si accumulation in panicles; affected Si distribution; impaired intervascular transfer; reduced grain Si content	Likely enhances Si distribution to shoots and panicles	Rice (*OsLsi6*/*OsNIP2;2*), Barley (*HvLsi6*), Maize (*ZmLsi6*), Finger millet (*EcLsi6*)	Two NPA motifs; similar selectivity to Lsi1; distributed expression pattern; critical for panicle Si accumulation	[95,167]

OsNIP3;2	NIP; NIP-III	Influx (As(III) uptake)	Lateral roots, stele region of primary roots	Plasma membrane	As(III) uptake in lateral roots; minor role in shoot As accumulation	Decreased As in roots; little effect on shoot As; limited contribution to As translocation	Not well characterized	Rice (*OsNIP3*;2)	NIP-type; high As(III) permeability in oocytes; expression suppressed by high As(III)	[168,169]
EcLsi1	NIP; NIP-III	Influx (Si uptake)	Roots; leaf sheaths & blades (higher expression)	Plasma membrane; predicted membrane localization	Si influx; co-evolved with EcLsi2; induced upon Si amendment	Transcript increased upon Si amendment (72 hpi)	Enhanced Si uptake	Finger millet (*EcLsi1*), Ryegrass (*LpLsi1*)	NIP2 subfamily; bona fide Si transporter; co-evolved with *EcLsi2*	[170]
EcLsi2	Anion transporter; ArsB-like transporter	Efflux (Si loading)	Roots; shoot tissues	Plasma membrane; predicted membrane localization	Si efflux; co-evolved with EcLsi1; upregulated in response to stress	Transcript increased upon Si amendment; upregulated upon stress (72 hpi)	Enhanced Si loading into xylem	Finger millet (*EcLsi2*), Cucumber (*CsLsi2*), Ryegrass (*LpLsi1*)	ArsB-type; distinct from *EcLsi1*; co-evolved during evolution	[162,171]
EcLsi3					Si efflux; derived through fusion/duplication; altered upon Si amendment	Transcript altered upon Si amendment	Enhanced Si loading	Finger millet (*EcLsi3*)	ArsB-type; evolved from *EcLsi1*/*EcLsi6* by duplication /fusion	[171]
GmNIP2.1 GmNIP2.2	NIP; NIP-III	Influx/distribution	Shoot tissues (induced under salt stress)	Plasma membrane	Induced under salt stress; involved in Si distribution & contributes to salt tolerance	Upregulated under salt stress	Enhanced salt stress tolerance	Soybean (*GmNIP2.1*) & (*GmNIP2.2*)	Plasma membrane localized; stress-responsive expression	[172]

Table S2. Main mechanisms by which Si mitigates the toxicity of selected metal(loid)s in soil-plant systems.

Element	Mechanism category	Level of action	Mechanism description	Key processes involved	Outcome on metal behavior	Outcome on plant response	Crops	Si source/ application mode	Reference
Cd	Uptake & transport inhibition	Root	Si forms barriers in cell walls and beneath cuticle; competes with Cd for transport pathways via Lsi1/Lsi2 transporters; reduces transporter gene expression	Si transporter activity (Lsi1, Lsi2); monosilicic acid uptake; cell wall polymerization; downregulation of NRAMP5, HMA2, HMA4 transporters	Reduced Cd^{2+} influx (35.57-42.30%); decreased Cd accumulation in roots (34.03%); reduced bioavailability	Improved root growth; enhanced root morphology; reduced Cd toxicity symptoms; improved nutrient uptake	Rice	Sodium silicate (Na_2SiO_3); soil application 100-200 kg SiO_2 ha^{-1}	[3]
	Root immobilizatio n / compartmenta tion	Root tissue/ cellular	Si mitigates Cd toxicity by reducing Cd uptake/accumulation in roots and promoting Cd compartmentation in root tissues.	Reduced Cd entry into roots; increased CAT, SOD, POD and proline; lower H_2O_2 and MDA; improved nutrient status.	Cd accumulation in roots reduced by 38% and 22% with 1 mM Si, and by 52% and 39% with 3 mM Si under 50 and 200 μM Cd, respectively	Improved root biomass and morphology; lower oxidative stress; better nutrient balance under Cd stress	Wheat	Si supplied in nutrient solution as silica gel derived from sodium silicate (Na_2SiO_3), 1 or 3 mmol L^{-1}	[173]
	Phytolith-based sequestration	Tissue/ Ecosystem	Si polymerizes to form phytoliths (SiO_2) that immobilize metals as phytolith-like particulates (e.g., $CdSiO_3$); provides long-term sequestration	Bio-silicification process; phytolith formation in cell walls & vacuoles; incorporation of metals into silica matrix; hydroxamate siderophore enhancement	Formation of stable $CdSiO_3$ particulates; 46.4% reduction in grain Cd; 41.2% increase in stem Cd; long-term sequestration	Reduced grain toxicity; enhanced food security; improved yield; prevented Cd remobilization	Rice	Si fertilizers; hydroxamate siderophores; field application	[174]
	Antioxidant defense enhancement	Cellular/ Biochemical	Si stimulates synthesis & activity of antioxidant enzymes (SOD, CAT, APX, GR); enhances non-enzymatic antioxidants (GSH, AsA); upregulates stress-responsive genes	Enzyme activation: SOD (+15.34-38.33%), CAT (+60.78%), APX, GR; ascorbate-glutathione cycle upregulation; gene expression of ROS-scavenging enzymes	Reduced H_2O_2 (11.29-21.88%); reduced MDA (oxidative damage marker); decreased O_2^- radicals; enhanced ROS scavenging capacity	Reduced oxidative stress; maintained chlorophyll content; enhanced photosynthesis; improved growth vigor; better membrane stability	Wheat Camelina, Wild rice	Silica gel; foliar spray 2.5-5 ppm; soil application	[175,176]
	Cell wall modification	Tissue	Si increases pectin, hemicellulose, lignin content; pectin's	Increased pectin & hemicellulose synthesis; pectin methylesterase (PME)	Cd sequestration in cell wall >soluble fraction; 2-3 fold	Reduced cytoplasmic Cd toxicity; maintained cellular	Pea, Tomato	Si foliar spray; seed priming with SiO_2NPs	[177,178]

			galacturonic acid binds Cd cations; strengthens structural integrity; enhances PME activity	activation; electrostatic bonding between carboxyl groups & Cd^{2+}	increased binding capacity; immobilization in apoplast	structure; improved water relations; enhanced stress tolerance			
	Photosynthetic enhancement	Physiological	Si maintains PSII efficiency, chlorophyll synthesis, & restores electron transport chain function; enhances stomatal conductance & CO_2 assimilation	Chlorophyll biosynthesis pathway activation; PSII structure stabilization; Rubisco activity restoration; photosynthetic gene upregulation	Reduced photo-inhibition; maintained electron transport; increased photosynthetic rate; reduced chlorophyll degradation	Net photosynthetic rate +14.21%; stomatal conductance +32.14%; leaf chlorophyll +37%; enhanced energy production for growth	Camelina, Wheat, Rice	Foliar Si spray; hydroponic Si supply 1.5-3 mM	[4,179,180]
	Osmolyte & secondary metabolite synthesis	Cellular	Si promotes proline, soluble sugar, & phenolic compound accumulation; regulates osmotic potential; enhances stress signaling molecules	Proline biosynthesis pathway activation; polyol synthesis enhancement; phenolic compound production via phenylpropanoid pathway	Increased osmotic adjustment; enhanced cellular turgor; improved water retention; maintained osmotic balance	Better osmotic regulation; enhanced turgor maintenance; improved growth under stress; enhanced phenolic defense compounds	Maize, Fenugreek	Si foliar spray 4 mM; combined Si + strigolactone application	[5,181]
**As**	Transporter competition & regulation	Root	Si competes with AsV for phosphate transporters (OsPT8) & with AsIII for aquaporin/NIP channels; silicic acid transporter pathway involved	Competitive inhibition of arsenate transporters; downregulation of As(III) transporter genes; Lsi-mediated As transport modulation	Reduced As bioavailability in rhizosphere; decreased As uptake 20-30%; reduced translocation to shoots	Lower tissue As accumulation; reduced As toxicity symptoms; improved plant growth; enhanced nutritional quality	Rice, Wheat, tomato	Seed priming with Si; Na_2SiO_3 soil application 20 mg kg^{-1}	[182,183]
	Metal-nanoparticle complex formation	Rhizosphere/ Tissue	Si nanoparticles form As(III)=SiNPs complexes in soil & tissues; immobilizes As preventing bioavailability; induces phytochelatin synthesis	SiNPs-As(III) complex formation; hydrogen bonding & chelation interactions; root exudate-phytochelatin (PC) formation; As sequestration	Soil As(III) immobilization; 40-50% increase in plant growth; reduced As bioavailability; As sequestration in biomass	Tripled yield weights; 40-50% growth increase; accelerated flowering & fruiting; improved food safety	Maize	Green synthesized SiNPs (100% v/v soil incorporation); SiNPs from *Pycreus polystachyos*	[184]
	Oxidative stress mitigation	Cellular/ Biochemical	Si nanoparticles serve as co-enzymes activating antioxidant systems;	SiNPs act as enzyme co-factors; enhanced SOD, POD, CAT activity; increased glutathione pools; above 70%	Reduced H_2O_2 24-32%; reduced O_2^- 29-36%; decreased MDA	Lower oxidative stress markers; maintained membrane integrity;	Rapeseed, Soybean, Wild rice	SiO_2NPs seed priming; Si nanoparticles foliar application 50 µM	

			enhance ROS scavenging; reduce oxidative damage	ROS scavenging (OH radicals)	content; reduced membrane damage	improved photosynthetic capacity; enhanced plant vigor		
	Photosynthetic recovery	Physiological	Si nanoparticles restore chloroplast structure & PSII efficiency; maintain photosynthetic enzyme activity; enhance light-capture capacity	PSII structure stabilization; chlorophyll synthesis enhancement; photosynthetic antenna protein regulation; electron transport restoration	Reduced photo-inhibition; maintained photochemical efficiency; increased photosynthetic rate; enhanced light energy conversion	Improved shoot & root fresh weight (+22.4-29.9%); enhanced chlorophyll & carotenoid content; improved biomass allocation	Rice	SiO_2NPs application 50-100 µM hydroponically	[185,186]
**Pb**	Physical barrier formation	Tissue	Si accumulates beneath leaf cuticle & in cell walls creating mechanical barriers; reduces Pb penetration; enhances structural integrity	Si deposition in waxy cuticle layer; cell wall polymerization; silica matrix formation; mechanical strengthening	Reduced Pb translocation to aerial parts 27%; decreased Pb in upper plant tissues; immobilization in roots	Reduced Pb toxicity symptoms; improved foliar health; maintained chlorophyll; enhanced growth parameters	Rice, Cabbage	SiNPs (2.5 mM) + Fe-NPs (25 mg L^{-1}) combined application; foliar Si spray 2 mM	[187]
	Antioxidant system enhancement	Cellular	Si combined with other nutrients (Fe, Zn) upregulates SOD, POD, CAT, GR; enhances glutathione pools; coordinates enzymatic & non-enzymatic defenses	Upregulation of antioxidant enzyme genes; enhanced synthesis of SOD (+114%), POD (+186%), CAT (+135%), GSH (+151%)	Reduced H_2O_2 & oxidative damage markers; enhanced ROS scavenging; maintained redox balance	Lower MDA content; improved photosynthetic efficiency; enhanced growth vigor; maintained cellular integrity	Fenugreek	Combined Si (2 mM) + 24-EBL (10-7 M) foliar application	[188]
	Morpho-physiological recovery	Physiological	Si-Fe nanoparticles restore growth parameters through improved nutrient uptake & stress tolerance; enhance biomass allocation	Nutrient translocation improvement; growth hormone balance restoration; biomass accumulation pathway activation	Reduced Pb uptake; enhanced nutrient availability; improved stress hormone balance	Shoot length +40%; shoot fresh weight +48%; root fresh weight +31%; improved flowering & reproduction	Rice	Si-Fe nanoparticles co-application: SiNPs (2.5 mM) + Fe-NPs (25 mg L^{-1}) hydroponic supply	[187,189]
**Cr**	Mechanical barrier formation	Tissue	Si deposits beneath cuticle & in cell walls; creates physical impediment to Cr influx; strengthens epidermal structure	Silica polymerization in cell wall matrix; cuticle thickening; hydrophobic barrier formation; mechanical strengthening	Reduced Cr translocation to shoots; decreased Cr concentration in leaves (-52.8%),	Reduced Cr toxicity symptoms; maintained leaf color & turgor; improved	Freesia, Sage	Si-NPs (10 mM) + IBA (20 mM) foliar application	[190]

					flowers (-78.5%); maintained rhizosphere barrier	photosynthetic efficiency			
	Comprehensive antioxidant defense	Cellular/ Biochemical	Si nanoparticles enhance enzymatic antioxidants (POD, CAT, SOD) & non-enzymatic defenses; maintains glutathione & ascorbate pools	Enhanced POD (+30.8%), CAT (+52.4%), SOD (+60.8%) activity; glutathione cycle upregulation; ascorbate synthesis enhancement	Reduced H_2O_2 (-32.7%); reduced MDA (-54.3%); maintained oxidative enzyme activity; enhanced protective protein pools	Improved growth morphology; enhanced leaf chlorophyll (+30.3%), carotenoid (+57.2%); vase life extension (+34.9%)	Freesia		
	Photosynthetic restoration	Physiological	Si supports photosynthetic antenna proteins & electron transport; maintains stomatal conductance; enhances light capture	Photosynthetic antenna protein regulation (CAB13, CAB6A); stomatal conductance modulation; photosynthesis-related gene expression	Maintained photosynthetic rate; enhanced intercellular CO_2 concentration; preserved light capture efficiency	Intercellular CO_2 concentration (+33.3%); stomatal conductance (+25.6%); photosynthetic rate (+31.1%); improved energy production	Freesia, Ornamental crops	Si nanoparticles foliar spray; combined Si + plant hormones	
**Ni**	Competitive Inhibition of Uptake	Root	Si competes with Ni for root uptake; strengthens root architecture; enhances selective nutrient uptake; reduces symplastic Ni flow	Root morphology improvement; cell wall barrier formation; nutrient transporter competition; reduced nickel transporter activity	Reduced Ni accumulation in roots (-67%); decreased Ni in leaves (-72%); improved nutrient (N, P, K) uptake	Enhanced root length & density; improved shoot growth; reduced Ni toxicity symptoms; better nutrient status	Cabbage	Foliar Si application 2 mM	[138]
	Osmolyte & antioxidant upregulation	Cellular	Si enhances proline & glycine betaine accumulation; upregulates antioxidant enzyme activities; improves osmotic adjustment	Proline biosynthesis pathway; glycine betaine synthesis enhancement; SOD, CAT, POD activity upregulation; glutathione cycle activation	Improved osmotic potential; enhanced cellular turgor; reduced oxidative stress; maintained redox balance	Improved shoot & root morphology; enhanced growth vigor; better water relations; reduced wilting			
**Al**	Root morphology & architecture enhancement	Tissue/ organ	Si strengthens root cell walls & promotes deeper, more prolific root development; reduces Al-induced root inhibition;	Cell wall strengthening via silica deposition; lignin & hemicellulose enhancement; lateral root primordium development	Reduced Al-induced root growth inhibition; enhanced root hydraulic conductance;	Deeper root systems; increased lateral roots; improved water stress tolerance; enhanced nutrient acquisition	Eucalyptus	Si application 0.5-1.5 mM solution ; Si fertilizer soil application	[191]

			enhances hydraulic conductance		improved water & nutrient uptake				
	Coordinated antioxidant defense	Cellular/ Biochemical	Si synergistically enhances enzymatic & non-enzymatic antioxidants; upregulates ROS-scavenging pathways; maintains redox homeostasis	SOD, CAT, APX activity synergistic increase; ascorbic acid & glutathione pool enhancement; glutathione peroxidase activation	Reduced ROS generation; enhanced ROS scavenging; reduced lipid peroxidation; maintained oxidative enzyme activity	Reduced oxidative stress markers; improved plant growth quality; enhanced tolerance; phytoremediation rate +18.7-34.8%			
**Zn**	Nutrient homeostasis maintenance	Cellular	Si combined with Zn establishes competitive uptake mechanisms; dilutes toxic Zn through improved plant biomass; enhances nutrient balance	Competitive transporter inhibition; enhanced macronutrient uptake; dilution effect through improved plant growth; selective ion absorption	Reduced Zn toxicity through competitive inhibition; maintained essential element balance; improved nutrient allocation	Enhanced plant growth; improved nutrient status; reduced Zn-induced chlorosis; better stress tolerance	Rice	Combined Zn (10 μM) + Si (15 μM) hydroponically	[192]
	Photosynthetic capacity recovery	Physiological	Si restores PSII efficiency & chloroplast structure; maintains chlorophyll synthesis; enhances photosynthetic gene expression	Chlorophyll biosynthesis pathway activation; PSII structure stabilization; photosynthesis-related gene upregulation (PsbY, PsaH, PetC, PetH)	Restored photosynthetic parameters; maintained intercellular CO_2; enhanced electron transport; reduced photo-inhibition	Enhanced photosynthetic rate; maintained chlorophyll fluorescence; improved light capture; better energy production	Oleander	Zn 0.76 μM + Si 0.5 mM	[193]
